# Radiographic False Evidence of a Tibial Baseplate Fracture After Total Knee Arthroplasty

**DOI:** 10.1016/j.artd.2021.02.004

**Published:** 2021-03-08

**Authors:** David S. Constantinescu, Jeremy A. Ross, Nirav K. Patel, Benjamin M. Strong, Laura A. Giambra, Gregory J. Golladay

**Affiliations:** a Department of Orthopaedic Surgery, University of Miami, Miami, FL, USA; b Department of Orthopaedic Surgery, VCU Health, Richmond, VA, USA; c Orthopaedic Associates of Michigan, P.C. Grand Rapids, MI, USA

**Keywords:** Implant failure, Knee arthroplasty, Complications

## Abstract

Fracture of the tibial baseplate is a rare but dramatic cause of typically late fatigue failure in the setting of loosening after total knee arthroplasty. A 58-year-old female presented 4 months after total knee arthroplasty for evaluation of contralateral knee pain. Plain radiographs of the left knee incidentally suggested the possibility of tibial baseplate fracture despite minimal, expected postoperative symptoms. Subsequent computed tomography imaging demonstrated no confirmatory evidence of component failure or fracture. Malalignment and fatigue fracture are proposed etiologies of baseplate fractures. The presented case illustrates the importance of computed tomography imaging and clinical correlation when a diagnosis of baseplate fracture is suspected to avoid an unnecessary revision surgery.

## Introduction

Fracture of the tibial baseplate is a rare but potentially devastating complication after total knee arthroplasty (TKA) [1]. Patients often present with pain, and subsequent imaging demonstrates evidence of baseplate fracture [2]. Fatigue-induced failure is a proposed mechanism for TKA component failures [3,4]. Early revision arthroplasty should be considered as delay may compromise bone stock and can lead to metal debris generation. A recent publication reported mid-term catastrophic failure of a highly porous modular tibial component [5]. Thus, catastrophic implant failure should be in the differential for a painful TKA at any time point.

We report a case of radiographic imaging suggestive of possible tibial baseplate fracture in an otherwise minimally symptomatic female who had undergone TKA with a modern cementless implant at another institution 4 months before presentation. This case illustrates the importance of additional confirmatory imaging and clinical correlation when a diagnosis of baseplate fracture is suspected.

### Case history

A 58-year-old female who had undergone left TKA at an outside facility 4 months prior presented to our institution because of a change in insurance for follow-up of her left TKA and also for evaluation for right TKA. Her initial TKA was performed with modern cementless implants (Zimmer Nex Gen TM modular CR; ZimmerBiomet, Warsaw, IN). She had no perioperative or postoperative complications.

She reported mild (3/10) intermittent anteromedial left knee pain, swelling, and stiffness that she felt was gradually improving. She denied significant mechanical symptoms or startup pain. On the right knee, she had constant, severe (8/10), medial, and anterior knee pain, swelling, catching, and feelings of instability. Her symptoms were worse with sit-to-stand activities, stairs, and lateral movement. She also reported night pain. She had previously been treated with conservative measures including medications, injections, supervised physical therapy, and self-imposed weight loss.

She had a past medical history of hypertension, glaucoma, and thrombocytopenia. Physical examination demonstrated a height of 170 cm, weight of 99.3 kg, and calculated body mass index of 34.32 kg/m^2^. The left knee examination demonstrated a well-healing surgical scar with minimal effusion and no significant tenderness to palpation over the proximal tibia. She had passive range of motion of 0 to 115 degrees with an extension lag of less than 5 degrees. She had <5 mm of symmetric varus-valgus laxity at 30 degrees and 5 mm of anteroposterior translation with the knee flexed 90 degrees. The right knee examination demonstrated intact skin with a small effusion, range of motion 10-105 degrees with no extensor lag, a partially correctable varus deformity, medial tenderness, <5 mm of mediolateral laxity, and 5 mm anteroposterior laxity. Neurovascular examination of both knees was normal.

**Figure 1:**
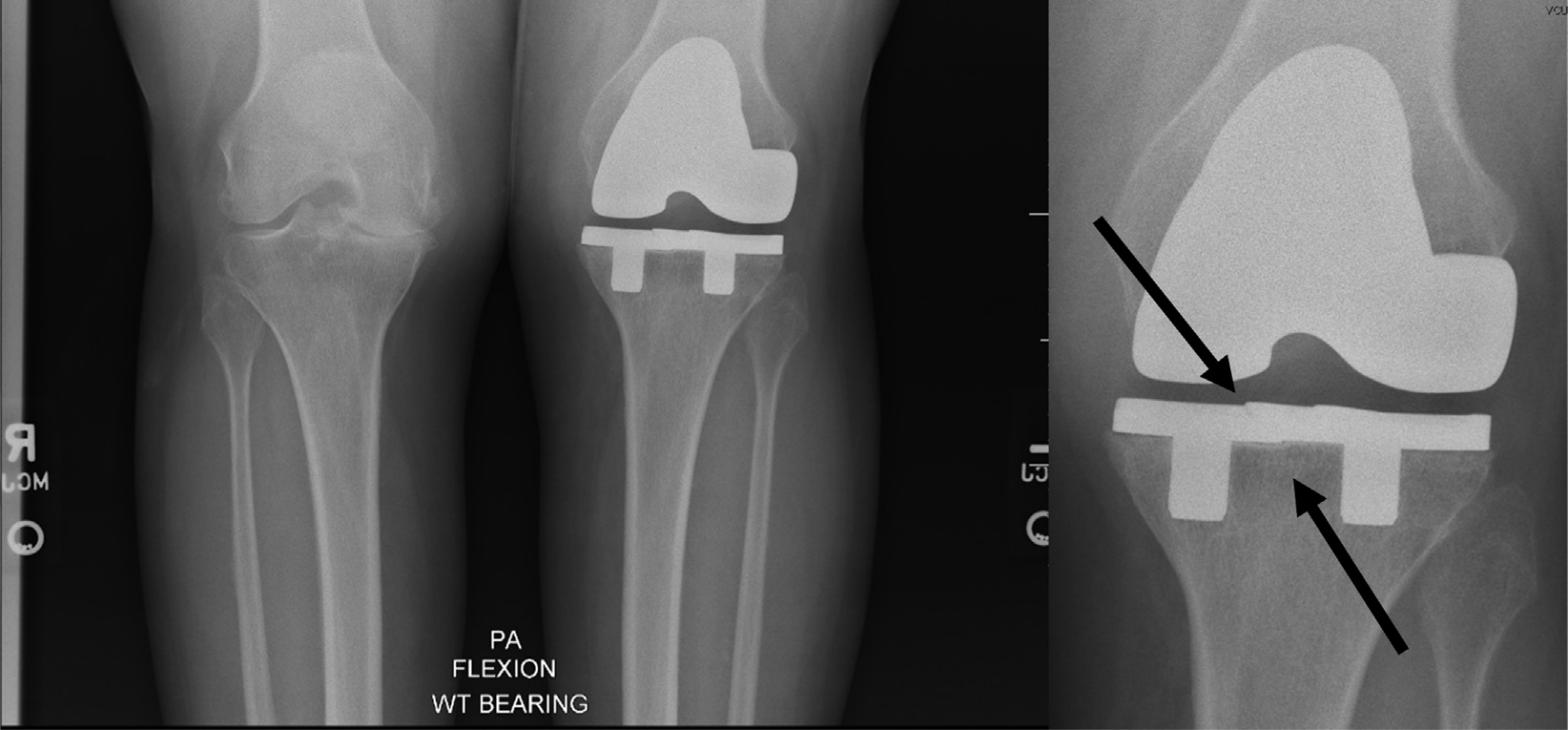
Standing posteroanterior flexion radiograph suggestive of tibial baseplate fracture (indicated by black arrows).

Radiographs of the left knee revealed the presence of a fully cementless TKA with overall good alignment and appropriate sizing. On the standing posteroanterior flexion view, there was a linear lucency just medial to the tibial prosthesis midline, suggestive of a possible fracture of the tibial baseplate (Fig. 1). These findings were confirmed by the musculoskeletal radiologist and included in their final report impression.

The primary surgeon was contacted to discuss the findings, and a computed tomography (CT) scan was obtained to better evaluate for possible tibial baseplate fracture. Additional workup also included serology and left knee arthrocentesis. Erythrocyte sedimentation rate (ESR) was 24 mm/h (normal range: 0 – 20), and C-reactive protein (CRP) was 0.5 mg/dL (normal range: 0 – 0.5). Cell count was 270 white blood cells/mm^3^ with 45% polymorphonuclear leukocytes. Joint fluid culture was negative.

**Figure 2:**
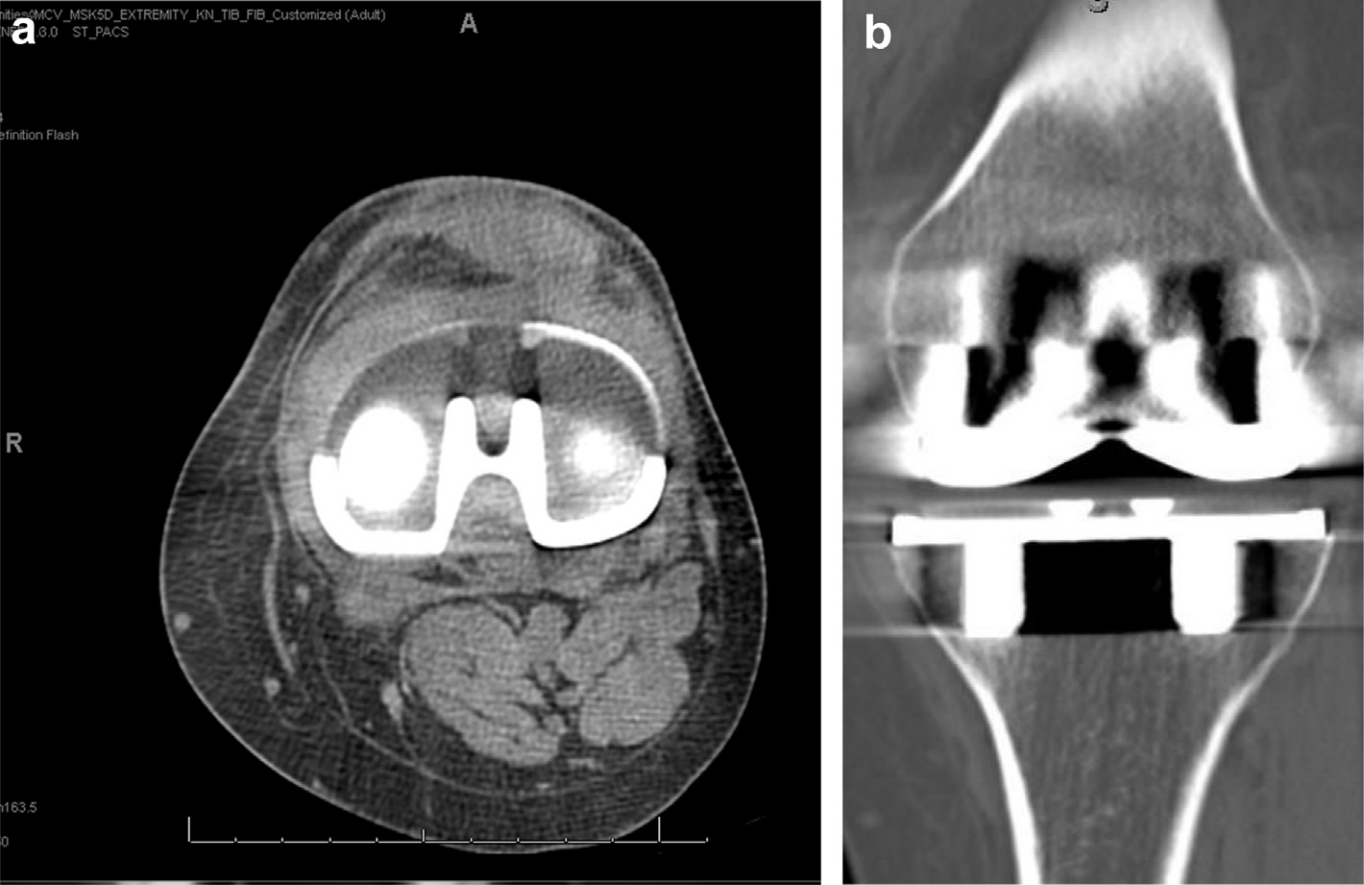
(a) Axial CT scan demonstrating intact tibial baseplate. (b) Coronal CT scan demonstrating intact tibial baseplate.

The patient presented for follow-up 3 weeks later with subjective improvement in left knee pain and was overall pleased with her continued recovery. She denied any symptoms of instability, and her physical examination was unchanged from the prior visit. The CT scan showed acceptable positioning of the tibial component with no evidence of baseplate fracture (Fig. 2).

Discussion with the patient occurred regarding the lack of definitive evidence of a tibial baseplate fracture as the initial films had suggested. The plain radiographic finding was assumed to be due to image projection related to the polyethylene locking mechanism. Given her asymptomatic presentation and lack of definitive imaging findings, revision surgery was not pursued.

**Figure 3:**
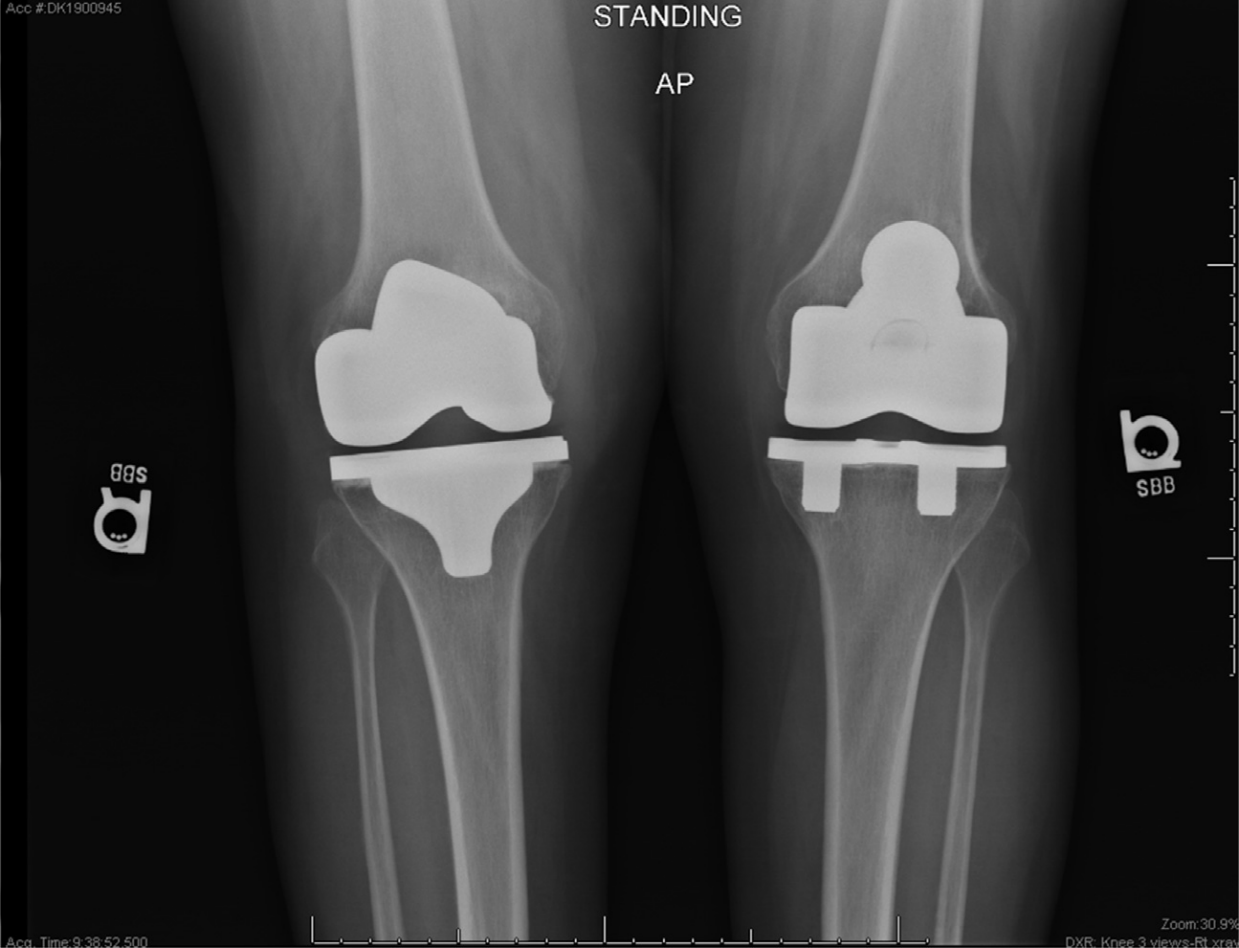
Standing anteroposterior radiograph 8 months after operation with no evidence of tibial baseplate fracture.

The patient then underwent right TKA and had an uneventful perioperative and postoperative course. Standard postoperative clinical and radiographic monitoring included assessment of her left TKA. At 8-month follow-up from her left knee operation, radiographs demonstrated a well-aligned TKA and intact tibial baseplate on the left and acceptable component sizing and alignment and fixation on the right (Fig. 3).

Informed consent was obtained from the patient for publication of her clinical case.

## Discussion

Tibial baseplate fractures are a rare complication after TKA. Management of an encountered baseplate fracture centers around early revision [1]. Delayed diagnosis may be a contributing factor to poor outcome because of bone loss that can occur from the altered loading environment of the knee with implant fracture [6]. Complete radiographic workup is required for definitive diagnosis including advanced imaging with CT scan. Early baseplate fractures have historically been attributed to poor surgical technique, implant manufacturing issues, or poor bone quality [6]. Proposed mechanisms leading to late baseplate fractures include malalignment and fatigue failure, bone loss due to osteolysis, undersizing, and elevated patient weight. [3,4,7].

In our presented case, the short duration between implantation and presentation effectively eliminated many known etiologies for implant fracture from possibility. Sizing of the implant appeared appropriate, and the patient’s body mass index (34) is not outside of the typical range seen in patients undergoing TKA at most centers. Of note, this implant is designed for cementless fixation but in fact is not approved for this indication by the Federal Drug Administration. Cementless fixation has been implicated as an additional cause of failure. A defect during manufacturing of the implants may also lead to early failure due to suboptimal processing. These factors were considered most likely while appropriately working the patient up for tibial baseplate fracture. Ultimately, implant failure could not be confirmed by additional advanced imaging or other appropriate diagnostic studies.

Our case report presents a diagnostic dilemma and an unusual treatment challenge. While the concerning finding was only present on a single projection, further workup and continued monitoring was necessary, given both the risk of failure and the potentially catastrophic consequences of treatment delay in the event of a broken tibial component. Fortunately, the patient’s clinical course, serial radiographs, and initial CT have shown no confirmatory evidence of baseplate fracture, and she is now under routine longitudinal surveillance. This case highlights the importance of having a high index of suspicion for implant complications and performing the appropriate diagnostic techniques to confirm or disprove the diagnosis. It is also imperative to have an understanding of potential etiologies for such failures to perform the necessary workup. If suspicion is raised for artifactual projection leading to implant fracture, repeat films with an oblique view in office is one option to consider. If fluoroscopy is available, this may be used as another method to quickly obtain alternate views. Furthermore, a 3D understanding of the implants used can aid in delineating how an illusion of a contiguous fracture can result.

## Summary

Fracture of the tibial baseplate is a rare but potentially devastating complication after TKA. Early diagnosis is important to prevent delay in revision surgery. We present a case in which initial radiographs were suggestive of a tibial baseplate fracture, but a follow-up CT was nonconfirmatory. This highlights the importance of clinical correlation and advanced imaging in the workup to prevent unnecessary revision surgery.

## Conflict of interests

See the following blinded combined form or individual unblinded forms. Gregory J. Golladay, MD, FAOA serves as Editor in Chief of Arthroplasty. He was recused from the editorial decision on this manuscript, which underwent blinded peer review.

For full disclosure statements refer to https://doi.org/10.1016/j.artd.2021.02.004

## Appendix A Supplementary data

Conflict of Intetest Statement for All Authors
